# Vascular calcification; Stony bridge between kidney and heart

**DOI:** 10.34172/jcvtr.2020.29

**Published:** 2020-07-26

**Authors:** Behzad Zaker, Mohammadreza Ardalan

**Affiliations:** ^1^Kidney Research Center, Tabriz University of Medical Sciences, Tabriz, Iran; ^2^Department of Biological Sciences, School of Natural Sciences, University of Tabriz, Tabriz, Iran

**Keywords:** Kidney, Vascular, Chronic Kidney Disease

## Abstract

Vascular calcification is a high prevalent complication that arises as a consequence of impaired calcium and phosphate balance amongst cardiovascular patients. Multiple inducer/ inhibitory molecules and pathways as well as genetic background and lifestyle play role in this phenomenon. According to which vessel layer (intima, media or both) is involved different types of vascular calcification take place. Actual mechanism and consensus pathways have not been elucidated yet and needs further investigations.

## Introduction

Cardiovascular disease is the major cause of death and significantly prevalent in chronic kidney disease (CKD) patients. Vascular calcification (VC), the pathological calcification of cardiovascular system by accumulation of calcium phosphate crystal deposits in the medial and intimal layers of the vessel wall, is one of the major causes of morbidity and mortality around the world.^1^ This process was initially considered as a passive response to degenerative events such as overload of phosphate and calcium in circulation; however emerging evidence suggests that this procedure is an actively regulated form of matrix mineral metabolism and intracellular molecular processes that is regulated via a variety of molecular signaling pathways.^2^ VC is dysregulated under pathological status such as dyslipidemia, hypertension, and is a common problem among the elderly and those people suffering from CKD, type 2 diabetes mellitus, and atherosclerosis. Patients who suffer from VC are more susceptible to cardiovascular disease.

Hydroxyapatite (Ca_10_(PO4)_6_(OH)_2_) crystals are the main inorganic component of the bony tissue. These are secreted by osteoblastic vesicles and comprise the major bony tissue structure. Like osteoblasts, vascular smooth muscle cells (VSMCs), major cells correlated with medial calcification that normally reside in the media of blood vessels, are able to undergo differentiation to osteoblast-like cells and extrude matrix vesicles that contain proteins similar and some in common to osteoblastic vesicles. When these proteins are secreted by VSMCs, ectopic formation of bony tissue increases and results in VC.^3,4^

Although the main underlying common cause in different kinds of VC is deposition of hydroxyapatite crystals, evidence shows that variety of molecular pathways and biochemical reactions and also genetic predisposition contribute in pathogenesis of VC. In addition to disrupted metabolism and other pathologies that promote VC, genetics and hereditary predisposition is of great importance in genesis of VC. According to linkage analysis and association studies, multiple loci and genes have been linked to VC.^5,6^ In this review, we will review recent state of knowledge on different kinds of VC as well as genetic background and molecular pathways that are involved.

Patients with CKD exhibit accelerated calcification of the intima and media of the vessels. Traditional risk factors such as hypercholesterolaemia or arterial-hypertension fail to adequately explain this condition, pointing to an added role of non-traditional risk factors such as uremia, inflammation and oxidative stress and an imbalance of calcium and phosphate, lack of calcification inhibitors such as fetuin-A and Matrix Gla proteins (MGPs). So beyond this specific vascular medial calcification (calcific arteriosclerosis or Mönckeberg’s sclerosis) that happens in patients with long-standing CKD an accelerated calcification of intimal plaque (calcific atherosclerosis) may happens and accelerate the classical atherosclerosis in these individuals. Considering microvascular calcification disease and macravascular calcification as a definitive terms both of them happens with accelerated rate in CKD patients, as the first one is much more atherosclerosis related and the second one is more calcified arterial medial lesion.^7^

### Types of vascular calcification

Depending on which layer(s) of vessel wall (intima, media, or both) are engaged in VC, different types of VCs exist and each of which has its own subtypes.^3,8^ Histologically, VC can be categorized in two main types: *tunica intima* also known as atherosclerotic calcification that mineralization occurs in intima layer and *tunica media* or Mönckenberg’s sclerosis.^9^ It should be noted that distinction between these different types of VC based on type, anatomical location, histology, genetics, and molecular pathways is not simple. However, there are some common molecular and pathophysiological pathways between these two phenomena.^3,4^ Different forms of VC can occur due to loss of inhibitors and some result from gain of promoting factor, and some others cause by a combination of them or loss of equilibrium.^10^

### Vascular calcification regulatory factors

#### Bone morphogenetic proteins (BMPs)

BMPs are large group of proteins that can be found in variety of tissues and have fundamental role in development and organogenesis. These proteins are the largest group of proteins in the transforming growth factor (TGF-β) superfamily and perform their roles through phosphorylation and subsequent activation of some intermediate molecules that finally activate Smad transcription factors. MGP is an inhibitor of bone morphogenetic proteins (BMPs) that prevents VSMCs osteogenesis and calcification by antagonizing BMP-2 and BMP-4. These two proteins are involved in promoting calcification through TGF-β signaling pathway.^11,12^ Recent study by Wei et al, demonstrated that in comparison to normal aorta, mRNA expression and serum levels of both BMP-2 and BMP-4 and their receptor are upregulated in aorta lesions in rat model of CKD-related VC.^13^ In another study by Malhotra et al, using BMP signaling pathway antagonist in MGP deficient mice, VC rates decrease, indicating that BMP signaling is required for VSMC calcification.^14^

It has been shown that in contrast to aforementioned BMPs, BMP-7 have protective properties against calcification. Polymorphisms in *BMP* -7 gene are correlated with higher risk of calcification.^15^ In a series of studies, it has been shown that BMP-7 might inhibit VC and attenuates the severity of calcification by inhibition of TGF-β signaling pathway especially in high vitamin-D and phosphorous dosage.^16-18^

It is noteworthy to remind that mutations in *MGP* gene is also involved in a autosomal recessive condition called Keutel syndrome characterized by arterial calcifications, peripheral pulmonary stenosis, and cartilage calcifications.^19^

#### Vitamin K and warfarin

Vitamin K, as an essential cofactor, has an inhibitory impact on VC and exerts this effect through enzymatic γ-carboxylation of glutamic acid residues in MGP. Lack of vitamin K is common problem among CKD patients in comparison to control population.^20,21^ To accomplish γ-carboxylation, dietary vitamin K should undergo a reduction reaction by the function of an enzyme called vitamin K epoxide reductase (VKOR) to be activated. Warfarin blocks the reduction step and it has been shown that antagonizing vitamin K function by the use of warfarin promotes arterial calcification in rat models.^22^ In another study by Kaesler et al in uremic rats, a reduction in γ-carboxylase enzyme levels was seen that resulted in decreased rates of MGP.^23^

Alternatively vitamin K can inhibit calcification by activating a protein that is expressed in VSMCs called growth arrest-specific protein 6 (Gas-6). Mechanism of inhibition is mediated by preventing apoptosis in these cells. When these cells undergo apoptosis, their fragmentation particles serve as nests for calcium deposition and; consequently, promotion of calcification. Warfarin inhibits carboxylation of MGP and also activation of Gas-6 and enhances the calcification, so, it is recommended to use vitamin K supplementation in CKD patients and other diseases that are susceptible to calcification.^22,24^

#### Osteoprotegerin (OPG)

Osteoprotegerin (OPG) is a glycoprotein member of tumor necrosis factor (TNF) receptor family that is involved in regulation of bone mass/turnover and osteoblast/osteoclast formation.^25^ It plays role as a soluble receptor for TNF related apoptosis-inducing ligand (TRAIL) and receptor activator of NF-κB ligand (RANKL).^26^ Given to its interaction with and neutralizing apoptotic factor TRAIL, it is considered as an anti-apoptotic factor, therefore, OPG acts as anti-calcifying agent in this background.^26,27^ Studies show that OPG is the key regulator of bone remodeling by inhibition of interaction between RANKL, a specific receptor of RANK on cell surface. While the underlying molecular basis of signaling pathways in VC are not fully understood it has been shown that OPG deficient (OPG−/−) mouse models display calcification in renal arteries and aorta and also a reduction in bone mass accompanied by early onset osteoporosis.^28,29^

In a study by Zhou et al, they investigated the potential inhibiting role of OPG on VSMCs calcification through Notch1-RBP-Jκ signaling pathway. Their results showed that OPG down regulates the expression of MSX2, a target protein of the Notch1-RBP-Jκ signaling pathway, in VSMCs by blocking this pathway. Taken together, OPG is considered as a protective factor against VC due to its inhibitory effect on bone remodeling.^30^

#### Alkaline phosphatases and tissue nonspecific alkaline phosphatase

Alkaline phosphatases (APs) are plasma membrane outer side and matrix vesicles bound enzymes that are involved in monophosphate production from a broad range of substrates, which is the prerequisite element for bone mineralization and synthesis of hydroxyapatite.^31,32^ ALP is a proliferative phase marker for osteoblastic differentiation. The rate of calcification is increased by elevated levels of ALP activity in VSMCs. There are four different types of APs in humans three of which are homologous and tissue specific (intestinal, placental, placental-like) and are located at the end part of long arm of the human chromosome 2 (2q34-37). While the other one, called tissue nonspecific alkaline phosphatase (TNAP), is encoded by the ALPL gene located at chromosome 1 (1p36.1-p34) and is expressed in lots of tissues with the highest activity in kidney, liver, and bone.^33^ Except bone, physiological activity of AP in other tissues is not clear. In bone, it has been proven that a special AP isoenzyme (TNAP) participates in bone mineralization. Hydrolysis of pyrophosphate and creation of separate inorganic phosphates by the TNAP has two beneficial outcomes: (1) the rates of accessible local monophosphate rises and this phosphate incorporates with ca^2+^ to form hydroxyapatite, naturally occurring form of calcium apatite, and (2) the amount of pyrophosphate, a potent inhibitor of mineralization, declines.^32^

Genetic mutations in TNAP coding gene has been proven to participate in development of a genetic disease called hypophosphatasia (OMIM: 171760) that results in bone undermineralization and is indicative of the ALP role in calcification.^34^ Missense mutations in TNAP gene causes decreased or complete absence of ALP activity; leads to increased levels of TNAP substrates such as inorganic pyrophosphate (PPi), phosphoethanolamine, and pyridoxal-5’-phosphate. In the absence of ALP activity, these substrates and specially pyrophosphates accumulate and inhibit formation of hydroxyapatite and subsequent calcification.

Recent studies show that ectopic calcification due to overproduction of inorganic phosphate results from elevated levels of AP activity and leads to rare premature atherosclerosis and cardiovascular conditions such as Hutchinson-Gilford progeria syndrome or the generalized arterial calcification of infancy syndrome and also associates with more common situations like increased risk of cardiovascular events and mortality in the general population and higher coronary artery calcifications in hemodialysis patients. These data show that targeting ALP and PPi metabolic pathway could serve as a new effective target against ectopic mineralization.^35,36^

#### microRNAs

MicroRNAs (miRNAs, miRs) are small, noncoding, endogenous RNAs that regulate hundreds or thousands of genes transcription and translation post-transcriptionally. Epigenetic regulation of minas is involved in cellular and physiological functions of VSMCs such as proliferation, development, and metabolism.^37,38^ Broad range of studies has been performed about different kinds of miRs in VC. Recently, using miRNA profiling in an OPG deficient mouse model, it has been shown that during VC development miR30a, miR125b, and miR32 display higher expression while miR320, miR210, and miR29a expression reduced. Amongst them, miR32 through elevation of RUNX2 expression and PI3k signaling pathway activation was the most altered and active miRNA in VSMCs during VC progression and can be used as a possible novel diagnostic biomarker.^39,40^ Previously it was shown that miR204 binds to Runx2 (as a key transcription factor for osteoblastic differentiation) 3’-UTR-binding site and inhibits the expression and; subsequently, this knockdown leads to significant prevention of ALP activity and VC. Inhibition of miR204 resulted in opposite effects and elevates calcification. Altogether, these are indicative of miR204 as an endogenous reducer of Runx2 in VSMCs calcification.^41,42^

MiR34a is involved in several processes such as apoptosis, aging, and cancer cell proliferation.^43,44^ Through down-regulation of longevity-associated gene SIRT1 (sirtuin 1), miR34a can induce senescence. SIRT1 inhibits VC by prevention of VSMC senescence and subsequent calcification. Mir34a by targeting seed sequences of Axl 3’-UTR also inhibits anti-apoptotic receptor tyrosine kinase Axl, an anti-calcification gene, and prevents VSMC calcification.^45,46^ These results nominate mir34a as a calcification process promoter through interaction with SIRT1 and AXL and targeting of this microRNA may have therapeutic benefits.

Modulation of calcium deposition and mineralization rate has been shown with transfecting VSMCs by miR221 and miR222 mimics. Although *in vitro* studies demonstrated that miR221 and miR222 mimics transfection individually did not make any significant change in VSMCs calcification, transfection with a combination of them was effective significantly and so synergetic action of them is needed for elevation of calcium deposition rates and VSMCs trans-differentiation.^47^ They can increase calcium deposition through changing *Enpp1* and *Pit1* expression level and but not through Msx2 and Rnnx2, essential transcription factors in bone mineralization.^48^

### Genetics

VC etiology is multifactorial, comprised of complex interaction between environmental, genetic, and vascular factors. Different person to person calcification rates, even if the environmental factors and related pathologies are the same, is indicative of a pivotal role of genetics in pathogenesis of this condition.

#### Genome wide association studies

Several variants and risk alleles have been attributed to increase coronary artery calcification predisposition according to data released by GWAS catalog (https://www.ebi.ac.uk/gwas/efotraits/EFO_0004723). According to genome wide association studies, the most significant coronary artery calcification (CAC) related loci are 9p21, 6p24.1, 10q21.3, and 15q25.1.^49,50^ Among them, one SNP (rs1537370, P=2.3 × 10-^11^) in 9p21 locus has the strongest association with CAC and also myocardial infraction. This polymorphism is embedded in the vicinity of *CDKN2A* and *CDKN2B* tumor suppressor genes which are involved in cell cycle regulation and thus proliferation. The nearest gene to so called SNP is CDKN2B antisense RNA 1 (*ANRIL* ) which is involved in epigenetic regulation of numerous genes including *CDKN2A* and *CDKN2B* . Existence of polymorphism (rs1537370) causes increased ANRIL expression level and so a reduction in CDKN2A and CDKN2B levels and increased VSMC proliferation that may result in atherosclerosis and predisposition to coronary artery calcification.^5,51,52^

#### Monogenic Hereditary forms of calcification

#### Idiopathic infantile arterial calcification (IIAC)

Idiopathic infantile arterial calcification or generalized arterial calcification of infancy (GACI, OMIM 208000) is a heritable, rare autosomal recessive condition, characterized by extensive calcification of medium and large arteries such as renal arteries, aorta, and coronary arteries that is resulted from loss of function mutations in ecto-nucleotide pyrophosphatase/phosphodiesterase1 (ENPP1) gene. Death mostly occurs between the first day of life and first year, the average in 6 months because of myocardial ischemia or acute cardiac failure that happens as a result of coronary artery changes. Distribution between male and female is equal and also there is not ethnic preference.^6,53,54^

*ENPP* 1 gene is located on long arm of chromosome 6 (6q23.2) and encodes a protein called NPP1, an membrane bound enzyme that is responsible for conversion of extracellular ATP to AMP and inorganic pyrophosphate, as mentioned in above parts is an inhibitor of calcification and mineralization. Dysfunction or malfunction of NPP1 affects adenosine metabolism and limits PPi production and serum PPi rates which results in hydroxyapatite crystals growth and accelerates the calcification process.^55,56^

Recently by using next generation sequencing method Brunod et al, reported a novel homozygous mutation c.784A > G (p.Ser262Gly) in *ENPP* 1 gene in a male infant with GACI that suffered from extensive arterial calcifications. In second step, they tested the infant’s consanguineous parents and the result displayed the same mutation in a heterozygous manner, indicating an autosomal recessive pattern of inheritance.^57^ Until now, over 40 causative mutations that accounts for more than 75% of cases has been discovered in GACI patients.^53^

#### Calcification of joints and arteries (CALJA; OMIM 211800)

Calcification of joints and arteries mostly occurs due to recessive mutations in *NT5E* and is characterized by late onset VC of lower extremities. The gene Ecto-59-nucleotidase (*NT5E* ) is located on long arm of chromosome 6 (6q14.3). NT5E cooperates with ENPP1 in conversion and processing of extracellular ATP and nucleotides to membrane-permeable nucleosides. As mentioned, the first reaction of breaking ATP to AMP and PPi is carried out by NPP1 enzyme (product of *ENPP* 1 gene) and the second reaction which is conversion of AMP to adenosine and inorganic phosphate catalyzed by protein product of *NT5E* gene called CD73 that as the NPP1 is a membrane bound extracellular enzyme. Phenotypic manifestation of *NT5E* mutations is not as severe and restrictive as *ENPP* 1 mutations. Adenosine that is produced by the act of CD73 is an inhibitor of TNAP and reduces inorganic phosphate which is an inducer of calcification. Mutations in *NT5E* cause CD73 malfunction or dysfunction and consequently lower adenosine and higher production of Pi that leads to calcification.^5,55^

Correlation between NT5E and VC firstly was demonstrated by Hilaire et al in three families with symptomatic arterial calcification. Nine persons in these three families suffered from lower-extremities and joints calcification. Two causative mutations were reported, a homozygous nonsense mutation (c.662C→A) and a homozygous missense (c.1073G→A) in *NT5E* gene. One case with the same phenotypes was compound heterozygote for c.662C→A and c.1609dupA (p.V537fsX7) mutations. Cultured fibroblasts from patients harboring these mutations showed lower CD73 expression and activity. These results indicated that adenosine metabolism pathway disruption or malfunction contributes in ectopic calcification. Adenosine supplementations, TNAP inhibitors (lansoprazole), and adenosine-receptor agonists can be used as possible treatments.^58^

Second report on *NT5E* mutations in CALJA patients was released in 2015. In a Chinese family two novel mutations (c.1360G→A and c.1387C→T) that affects protein structure by changing the β-pleated sheet was discovered that leads to an impaired CD73 enzymatic activity.^59^

#### Pseudoxanthoma elasticum (PXE; OMIM 264800)

Pseudoxanthoma elasticum is a disease of elastin abundant in connective and soft tissues. It is characterized by dystrophic calcification of connective tissues including; arterial media, dermis, and the Bruch’s membrane of the eye. Mutations in *ABCC* 6 (ATP-binding cassette protein complex) that encodes a transmembrane protein-transporter has been related to the pathogenesis of PXE.^60^ *ABCC* 6 gene on the short arm of chromosome 16 (16p13.11) encodes trans-membrane protein–transported, MRP6 that is responsible for extracellular ATP transport and subsequent formation of ATP breakdown product PPi, an important mineralization inhibitor. Decreased hepatic production of calcification inhibitory factor of MGP that prevents BMP-2 activities is also related to ABCC6 malfunction.^4,61^

Genetic study of 3 patients with PXE phenotypes disclosed biallelic mutations in *ENPP* 1 gene which is responsible for GACI and on the other hand mutation screening of 28 patients with GACI showed *ABCC* 6 gene in 14 of them.^62^ Those overlaps shows that GACI and PXE are not two independent entities and actually they are two borders of calcification scope.^63^

## Conclusion

VC is a state of complex interaction between different role players, and any therapeutic consideration in this area needs in-depth understanding about its pathogenesis in any special situations. Different pathologies such as atherosclerotic calcification, arterial media sclerosis and heart valvular calcification may have overlapping and also yet distinct mechanisms. The prevalence of those pathologies are different based on special clinical situation, but end stage renal disease predispose the patient to be vulnerable to all those types.

Study of monogenic causes of VC not only helps their clinical diagnosis but also sheds light to more understanding about the path mechanisms of VC.

## Competing interests

None to declared.

## Ethical approval

Not applicable.

## Funding

No funding has been requested for this study.
